# Prevalence of Hypercalcemia in Patients With Pulmonary Tuberculosis and Impact of Treatment

**DOI:** 10.7759/cureus.100888

**Published:** 2026-01-06

**Authors:** Naina Naina, Kanwarpreet S Dhamija

**Affiliations:** 1 Internal Medicine, Government Medical College, Amritsar, IND; 2 Internal Medicine, Brar Multispecialty Hospital, Ludhiana, IND

**Keywords:** calcium metabolism, granulomatous disease, hypercalcemia, metabolic complications, tuberculosis, vitamin d

## Abstract

Background and aim

Hypercalcemia is a recognized metabolic complication in patients with pulmonary tuberculosis (TB), attributed to extrarenal synthesis of 1,25-dihydroxyvitamin D (1,25(OH)₂D or calcitriol) by activated macrophages within granulomatous lesions. Biochemical characterization, including parathyroid hormone (PTH) measurement, is essential for excluding alternative etiologies. Serum calcitriol measurement is critical for directly confirming the postulated mechanism. The aim of the study was to determine the prevalence, severity pattern, biochemical profile, including serum calcitriol, risk factors, and treatment response of hypercalcemia in adults with pulmonary TB.

Methods

Medical records of 100 adults with microbiologically confirmed pulmonary TB confirmed by sputum acid-fast bacilli (AFB) smear, mycobacterial culture (MC), or Mycobacterium tuberculosis rifampicin resistance (MTB/RIF) treated between January 1, 2022, and December 31, 2022, at Brar Multispecialty Hospital, Ludhiana, India, were reviewed. Corrected serum calcium was recorded at baseline, one, two, three, and six months. Hypercalcemia was defined as corrected calcium >10.5 mg/dL (2.62 mmol/L) and graded as mild (10.6-11.5 mg/dL), moderate (11.6-12.5 mg/dL), or severe (>12.5 mg/dL). All hypercalcemic patients underwent measurement of serum calcitriol (1,25-dihydroxyvitamin D), PTH, and 25-hydroxyvitamin D (25(OH)D) as per standard hypercalcemia evaluation protocol, with direct calcitriol measurement enabling confirmation of the granulomatous mechanism.

Results

Hypercalcemia (>10.5 mg/dL) occurred in 22 patients (22%; 95% CI: 14.6-31.6%) at baseline, with 16 (72.7%) classified as mild and six (27.3%) moderate. All hypercalcemic patients demonstrated suppressed PTH (6.2±2.0 pg/mL, range 3.3-9.6 pg/mL; reference range 10-65 pg/mL), confirming a non-parathyroid-mediated mechanism and excluding primary/secondary hyperparathyroidism. Serum 25(OH)D levels were within normal range (37.0±6.0 ng/mL; reference range 20-50 ng/mL). Serum phosphate was measured in all hypercalcemic patients with a mean of 3.1±0.5 mg/dL (reference range 2.5-4.5 mg/dL), supporting the granulomatous mechanism. Critically, direct measurement of serum calcitriol in all hypercalcemic patients revealed uniform elevation (78.5±15.2 pg/mL; reference range 15-65 pg/mL), directly confirming the extrarenal 1α-hydroxylase mechanism. Disseminated TB (multivariate OR: 2.4, 95% CI: 1.1-5.3, p=0.028) and chronic kidney disease (multivariate OR: 3.8, 95% CI: 1.3-11.1, p=0.015) were independent risk factors. Complete calcium normalization occurred in all hypercalcemic patients by six months of anti-TB therapy (median time 2.0 months, IQR: 1.3-2.9 months).

Conclusion

Hypercalcemia affects approximately 22% of patients with pulmonary TB. Suppressed PTH with normal 25(OH)D, normal phosphate, and elevated calcitriol confirms the characteristic biochemical pattern of granulomatous hypercalcemia resulting from extrarenal calcitriol synthesis. Complete resolution with anti-TB therapy supports calcium and vitamin D metabolite screening in high-risk TB populations.

## Introduction

Pulmonary tuberculosis (TB) remains a major global health challenge, with complex systemic manifestations extending beyond respiratory symptoms [1]. Among recognized metabolic complications, hypercalcemia has been documented since the early 20th century, though its prevalence, biochemical characterization, and clinical significance remain areas of active investigation [2,3].

The differential diagnosis of hypercalcemia encompasses both parathyroid hormone (PTH)-mediated and non-PTH-mediated etiologies [4]. Measurement of serum PTH represents the critical first-line investigation in hypercalcemia evaluation, as it distinguishes primary/secondary hyperparathyroidism (elevated or inappropriately normal PTH) from alternative causes, including malignancy, vitamin D disorders, and granulomatous diseases (suppressed PTH) [5,6]. In the normal physiologic state, hypercalcemia suppresses PTH secretion through calcium-sensing receptor activation on parathyroid chief cells, resulting in PTH levels below the reference range [7].

The pathophysiology of TB-associated hypercalcemia involves extrarenal synthesis of 1,25-dihydroxyvitamin D (1,25(OH)₂D or calcitriol) by activated macrophages within granulomatous lesions [8,9]. These activated macrophages express 25-hydroxyvitamin D-1α-hydroxylase (CYP27B1 gene product), converting circulating 25-hydroxyvitamin D (25(OH)D) to bioactive calcitriol in an unregulated manner, independent of parathyroid hormone and renal feedback regulation [10]. This mechanism, first rigorously demonstrated in anephric patients with sarcoidosis, results in a characteristic biochemical pattern: elevated serum calcium, suppressed PTH, normal or mildly elevated 25(OH)D, normal to low phosphate (reflecting PTH suppression), and markedly elevated calcitriol [11,12]. This biochemical profile effectively distinguishes granulomatous hypercalcemia from primary hyperparathyroidism (where phosphate is suppressed due to elevated PTH) and vitamin D intoxication (where 25(OH)D is elevated).

Reported prevalence rates of TB-associated hypercalcemia vary considerably across populations and methodologies. Contemporary investigations using albumin-corrected calcium measurements report rates between 15% and 51% [1,2]. Geographic variation has been documented: 25% in Swedish patients [3], 27.5% in Nigerian patients [13], and 20.15% in Indian populations [1]. While prior studies have documented TB-associated hypercalcemia [1-3] and examined vitamin D metabolism in bronchoalveolar lavage cells [10], comprehensive and simultaneous measurement of all four serum parameters, PTH, 25(OH)D, phosphate, and calcitriol, in hypercalcemic patients with TB distinguishes this investigation from prior work.

Clinical presentation ranges from asymptomatic biochemical abnormalities detected through routine screening (80-90% of cases) to symptomatic hypercalcemia with neuropsychiatric, gastrointestinal, renal, and musculoskeletal manifestations [1,2,13]. Current understanding indicates that TB-associated hypercalcemia typically resolves with anti-TB therapy [3], though systematic documentation of treatment response patterns remains limited.

The main aim of the study was to determine the prevalence and biochemical profile of hypercalcemia in adults with pulmonary TB. The secondary objective was to characterize hypercalcemia severity patterns, identify independent risk factors, and document temporal resolution with anti-TB therapy.

## Materials and methods

Study design and setting

This retrospective observational study was conducted at Brar Multispecialty Hospital, Ludhiana, India. The study protocol received approval from the Institutional Ethics Committee (approval number BMC/IEC/2022/007, approved on 07/01/2022), and patient confidentiality was maintained according to institutional guidelines.

Study population and timeline

We included 100 consecutive adults diagnosed with microbiologically confirmed pulmonary TB between January 1, 2022, and December 31, 2022. All patients were followed for a minimum of six months to assess treatment outcomes and calcium normalization patterns. All 100 enrolled patients had baseline serum calcium measurements available.

Inclusion criteria comprised adults aged 18 years or above with microbiologically confirmed pulmonary TB, defined as positive sputum acid-fast bacilli (AFB) smear (at least one of three specimens), positive Mycobacterium tuberculosis culture with drug susceptibility testing, or positive GeneXpert® (Cepheid Inc., Sunnyvale, US) Mycobacterium tuberculosis rifampicin resistance (MTB/RIF) assay. Additional requirements included radiological evidence of pulmonary involvement on chest radiography or computed tomography, completion of a full treatment course at the study institution, and availability of baseline and follow-up laboratory data, including serum calcium, albumin, and creatinine. For patients with hypercalcemia, the availability of parathyroid hormone, 25(OH)D, phosphate, and calcitriol measurements was required.

Exclusion criteria included previous TB treatment within preceding 12 months; multi-drug resistant tuberculosis (MDR-TB), defined as resistance to at least rifampicin and isoniazid, or extensively drug-resistant tuberculosis (XDR-TB), defined as MDR-TB plus resistance to fluoroquinolones and second-line injectable agents; exclusively extrapulmonary TB without pulmonary involvement; concomitant conditions affecting calcium metabolism including primary or secondary hyperparathyroidism, active malignancy, sarcoidosis, chronic kidney disease (CKD) requiring dialysis, and active thyroid disorders (hyperthyroidism or hypothyroidism); patients receiving medications affecting calcium metabolism: vitamin D supplements exceeding 1000 IU daily, calcium supplements exceeding 500 mg daily, thiazide diuretics, lithium therapy, or bisphosphonates.

Of 127 patients screened during the study period, 100 met the inclusion criteria and were included in the final analysis. During initial screening, 27 patients were excluded: 11 patients with MDR-TB and 16 patients receiving vitamin D and/or calcium supplementation exceeding predefined exclusion thresholds.

Sample size calculation

The sample size of 100 patients was determined to achieve 95% confidence in prevalence estimation with ±8% precision margin, based on an expected hypercalcemia prevalence of 20% reported in prior literature [1]. The observed prevalence of 22% falls within the calculated confidence interval, validating sample size adequacy.

Laboratory methods and definitions

Microbiological Confirmation Methods

Microbiological confirmation of PTB was performed by three methods: (1) sputum acid-fast bacilli (AFB) smear microscopy using Ziehl-Neelsen staining according to World Health Organization (WHO) standards; (2) Mycobacterium tuberculosis culture on Lowenstein-Jensen media with subsequent drug susceptibility testing using the proportion method; or (3) GeneXpert® MTB/RIF molecular assay for rapid detection of M. tuberculosis and rifampicin resistance. In this cohort, 62 patients (62%) were confirmed by sputum AFB smear positivity, 28 patients (28%) by positive culture, and 10 patients (10%) by GeneXpert® MTB/RIF assay.

Calcium Measurement and Correction

Hypercalcemia was defined as albumin-corrected serum calcium exceeding 10.5 mg/dL (2.62 mmol/L). Albumin correction was calculated using the standard formula: corrected calcium (mg/dL)=serum calcium (mg/dL)+0.8×(4−patient albumin g/dL). Severity classification followed established criteria: mild (10.6-11.5 mg/dL or 2.65-2.87 mmol/L), moderate (11.6-12.5 mg/dL or 2.90-3.12 mmol/L), severe (>12.5 mg/dL or >3.12 mmol/L). Calcium measurements were performed at baseline, one, two, three, and six months after treatment initiation.

Parathyroid Hormone and Vitamin D Metabolite Measurements

As per standard protocol for hypercalcemia evaluation, all patients with hypercalcemia underwent simultaneous measurement of serum PTH, 25-hydroxyvitamin D (25(OH)D), serum phosphate, and calcitriol (1,25-dihydroxyvitamin D or 1,25(OH)₂D). Serum PTH was measured using electrochemiluminescence immunoassay (ECLIA; Roche Cobas e411 analyzer, Roche Diagnostics, Mannheim, Germany) with reference range 10-65 pg/mL (1.1-7.0 pmol/L). Suppressed PTH was defined as values below 10 pg/mL. Serum 25(OH)D was measured using chemiluminescence immunoassay (DiaSorin S.p.A., Saluggia, Italy) with reference range 20-50 ng/mL (50-125 nmol/L). Serum phosphate was measured using the molybdate-based colorimetric method (Roche Cobas analyzer) with reference range 2.5-4.5 mg/dL (0.81-1.45 mmol/L). Serum calcitriol (1,25(OH)₂D) was measured using liquid chromatography-tandem mass spectrometry (LC-MS/MS; Waters TQD, Waters Corporation, Milford, US) with reference range 15-65 pg/mL (36-156 pmol/L).

The study was not designed to compare vitamin D metabolite levels between normocalcemic and hypercalcemic patients, as PTH, phosphate, 25(OH)D, and calcitriol were measured exclusively as part of standard diagnostic evaluation in patients with hypercalcemia.

Additional Definitions

Chronic kidney disease was defined as estimated glomerular filtration rate (eGFR) below 60 mL/min/1.73m² using the Chronic Kidney Disease Epidemiology Collaboration (CKD-EPI) equation, excluding patients requiring dialysis. Disseminated TB was defined as involvement of two or more non-contiguous organ systems (e.g., pulmonary plus lymph node, pleural, or meningeal involvement) based on clinical, radiological, and microbiological evidence.

Treatment protocol

All enrolled patients received standard first-line anti-TB therapy according to national TB elimination program guidelines: intensive phase with rifampin (10 mg/kg/day), isoniazid (5 mg/kg/day), ethambutol (15-25 mg/kg/day), and pyrazinamide (25-30 mg/kg/day) for two months, followed by continuation phase with rifampin, isoniazid and ethambutol at same doses for four months. Treatment adherence was monitored through directly observed therapy (DOT) for all patients and documented in clinical records. Of 100 patients, 93 patients (93%) completed the full six-month treatment regimen with less than 10% dose adherence gaps documented.

Statistical analysis

Statistical analyses were performed using Statistical Package for the Social Sciences (SPSS) version 28 (IMB Inc., Armonk, US). Continuous variables were assessed for normality using the Kolmogorov-Smirnov test. Normally distributed continuous variables were presented as mean±standard deviation and compared using Student's t-test. Categorical variables were summarized as counts and percentages, compared using Pearson's chi-square test or Fisher's exact test when expected cell counts were less than five.

For multiple statistical comparisons, the Bonferroni correction for multiple testing was applied. With eight variables compared between groups, the Bonferroni-corrected significance threshold was p<0.00625 (p=0.05/8).

Univariate logistic regression identified potential risk factors for hypercalcemia, with variables demonstrating p<0.05 entered into multivariate logistic regression analysis using a forward stepwise selection method. The multivariate model included disseminated TB and CKD as primary predictors. Results were expressed as odds ratios (OR) with 95% confidence intervals (CI). Prevalence estimates reported with exact binomial 95% confidence intervals. A two-tailed p-value less than 0.05 was considered statistically significant for primary analyses.

## Results

Demographics and baseline characteristics

The study cohort comprised 100 adults with microbiologically confirmed pulmonary TB, demonstrating male predominance with 66 males (66%) and 34 females (34%). Mean age was 43.2±14.8 years (range 18-79 years). Comorbid conditions were present in 38 patients (38%), including diabetes mellitus in 16 (16%), CKD (eGFR <60 mL/min/1.73m²) in nine (9%), human immunodeficiency virus (HIV) co-infection in seven (7%), and chronic obstructive pulmonary disease (COPD) in six (6%) patients. Nutritional assessment revealed undernutrition, defined as body mass index (BMI) below 18.5 kg/m², in 39 patients (39%), with a cohort mean BMI of 19.6±3.4 kg/m². Radiological evaluation demonstrated bilateral lung involvement in 62 patients (62%) and cavitary lesions in 34 (34%). Disseminated TB was identified in 17 patients (17%). Detailed baseline characteristics are presented in Table 1.

**Table 1:** Baseline characteristics and biochemical profile by calcium status Student's independent t-test was applied for continuous variables; Pearson's chi-square test for categorical variables. For multiple comparisons, the Bonferroni correction was applied with a corrected p-value threshold of p<0.00625 (eight variables compared). * significant after the Bonferroni correction (p<0.00625) ** highly significant (p<0.001) Parathyroid hormone (PTH) reference range: 10-65 pg/mL (suppressed if <10 pg/mL); 25-hydroxyvitamin D reference range: 20-50 ng/mL; serum phosphate reference range: 2.5-4.5 mg/dL; serum calcitriol reference range: 15-65 pg/mL PTH, 25(OH)D, phosphate, and calcitriol were measured only in hypercalcemic patients per hypercalcemia evaluation protocol ("-" not measured in normocalcemic group).

Parameter, mean±SD or n (%)	All patients (n=100)	Normocalcemic (n=78)	Hypercalcemic (n=22)	p-value
Age, years	43.2±14.8	42.8±15.1	44.7±13.8	0.457
Sex, male	66 (66.0)	50 (64.1)	16 (72.7)	0.436
Body mass index, kg/m²	19.6±3.4	19.8±3.5	18.9±3.1	0.123
Diabetes mellitus	16 (16.0)	11 (14.1)	5 (22.7)	0.328
Chronic kidney disease	9 (9.0)	4 (5.1)	5 (22.7)*	0.010*
Human immunodeficiency virus co-infection	7 (7.0)	5 (6.4)	2 (9.1)	0.641
Disseminated tuberculosis	17 (17.0)	10 (12.8)	7 (31.8)*	0.034*
Bilateral lung involvement	62 (62.0)	47 (60.3)	15 (68.2)	0.488
Cavitary lesions	34 (34.0)	24 (30.8)	10 (45.5)	0.199
Serum calcium, mg/dL	9.94±0.82	9.6±0.4	11.1±0.6**	<0.001**
Serum parathyroid hormone, pg/mL	-	-	6.2±2.0	-
Serum 25-hydroxyvitamin D, ng/mL	-	-	37.0±6.0	-
Serum phosphate, mg/dL	-	-	3.1±0.5	-
Serum calcitriol, pg/mL	-	-	78.5±15.2	-

Prevalence and characteristics of hypercalcemia

Hypercalcemia, defined as albumin-corrected calcium exceeding 10.5 mg/dL (2.62 mmol/L), was present at baseline in 22 patients, representing a prevalence of 22% (95% CI: 14.6-31.6%). Among these hypercalcemic patients, severity classification revealed 16 patients (72.7%) with mild hypercalcemia (10.6-11.5 mg/dL), six patients (27.3%) with moderate hypercalcemia (11.6-12.5 mg/dL), and no patients with severe hypercalcemia (>12.5 mg/dL). Mean serum calcium for the entire cohort was 9.94±0.82 mg/dL, with hypercalcemic patients demonstrating a mean of 11.1±0.6 mg/dL compared to 9.6±0.4 mg/dL in normocalcemic patients (p<0.001).

Clinical presentation and symptoms

The vast majority of hypercalcemic patients were asymptomatic (20 of 22, 90.9%), with only two patients (9.1%) presenting with symptoms. Symptoms assessed through a standardized checklist included nausea, polyuria, cognitive changes, headache, and constipation. Both symptomatic patients received intravenous isotonic saline with rapid calcium improvement and complete normalization within one month of anti-TB therapy. Details are presented in Table 2.

**Table 2:** Severity classification and clinical features of hypercalcemic patients (n=22) Hypercalcemia severity is classified by albumin-corrected serum calcium concentration. Symptomatic hypercalcemia was assessed using a standardized symptom checklist (nausea, polyuria, cognitive changes, headache, constipation). Student's t-test was used to compare calcitriol between mild and moderate hypercalcemia groups (p=0.008). Calcitriol reference range: 15-65 pg/mL. No patients with severe hypercalcemia were identified.

Severity category	Corrected serum calcium range, mg/dL	Frequency, n (%)	Symptomatic patients, n (%)	Calcitriol pg/mL, mean±SD
Mild	10.6-11.5	16 (72.7)	2 (12.5)	72.8±12.1
Moderate	11.6-12.5	6 (27.3)	0 (0)	91.3±14.7
Severe	>12.5	0 (0)	-	-

Biochemical profile of hypercalcemia

All 22 hypercalcemic patients underwent measurement of serum PTH, 25(OH)D, serum phosphate, and 1,25-dihydroxyvitamin D (calcitriol) as per standard hypercalcemia evaluation protocol. Serum PTH levels were uniformly suppressed in all hypercalcemic patients, with a mean of 6.2±2.0 pg/mL (range 3.3-9.6 pg/mL), compared to the reference range of 10-65 pg/mL. Notably, 100% of hypercalcemic patients had PTH values below 10 pg/mL, confirming suppression and effectively excluding primary or secondary hyperparathyroidism as the etiology of hypercalcemia.

Serum 25-hydroxyvitamin D levels in hypercalcemic patients were within the normal reference range, with a mean of 37.0±6.0 ng/mL (range 28-48 ng/mL; reference range 20-50 ng/mL). This normal 25(OH)D pattern excludes vitamin D intoxication and supports the mechanism of extrarenal 1α-hydroxylase activity converting normal circulating 25(OH)D to excessive calcitriol.

Serum phosphate levels in hypercalcemic patients were within normal range (3.1±0.5 mg/dL; reference range 2.5-4.5 mg/dL), with only two patients (9.1%) demonstrating phosphate at the lower end of normal (2.5-2.8 mg/dL). This normal-to-mildly-low phosphate pattern, in the setting of suppressed PTH, is consistent with the expected renal response to PTH suppression and differentiates granulomatous hypercalcemia from primary hyperparathyroidism, where significant phosphate suppression would be expected due to elevated PTH-mediated urinary phosphate wasting.

Critically, serum calcitriol levels were elevated in all hypercalcemic patients, with a mean of 78.5±15.2 pg/mL (range 58-112 pg/mL; reference range 15-65 pg/mL). This finding directly confirms the hypothesized pathophysiological mechanism of extrarenal 1α-hydroxylase (CYP27B1 enzyme) activity in activated macrophages within granulomatous lesions. The degree of calcitriol elevation was proportional to hypercalcemia severity: mild hypercalcemia (n=16) showed a mean calcitriol of 72.8±12.1 pg/mL, while moderate hypercalcemia (n=6) showed a mean calcitriol of 91.3±14.7 pg/mL (p=0.008).

Risk factor analysis for hypercalcemia

Univariate logistic regression analysis examined the following variables: age, sex, BMI, diabetes mellitus, CKD, HIV co-infection, disseminated TB, bilateral lung involvement, and cavitary lesions. Variables with p<0.05 identified in univariate analysis were disseminated TB (OR: 3.2, 95% CI: 1.1-9.4, p=0.034) and CKD (OR: 5.4, 95% CI: 1.4-20.6, p=0.010).

Multivariate logistic regression analysis, incorporating variables with p<0.05 from univariate analysis using forward stepwise selection, confirmed both disseminated TB (adjusted OR: 2.4, 95% CI: 1.1-5.3, p=0.028) and CKD (adjusted OR: 3.8, 95% CI: 1.3-11.1, p=0.015) as independent predictors of hypercalcemia. Risk factor data are detailed in Table 3.

**Table 3:** Risk factors for hypercalcemia - univariate and multivariate logistic regression analysis * significant at p<0.05 in univariate analysis ** significant in multivariate analysis Variables examined: age, sex, BMI, diabetes mellitus, CKD, HIV co-infection, disseminated TB, bilateral lung involvement, and cavitary lesions. Disseminated TB and CKD remained in the final multivariate model as independent predictors.

Risk factor	Hypercalcemic (n=22), n (%)	Normocalcemic (n=78), n (%)	Univariate OR (95% CI)	p-value	Multivariate OR (95% CI)	p-value
Disseminated tuberculosis	7 (31.8)	10 (12.8)	3.2 (1.1-9.4)*	0.034*	2.4 (1.1-5.3)**	0.028**
Chronic kidney disease	5 (22.7)	4 (5.1)	5.4 (1.4-20.6)*	0.010*	3.8 (1.3-11.1)**	0.015**

Treatment response and calcium normalization

All patients with baseline hypercalcemia achieved complete normalization of serum calcium during the anti-TB therapy course. Calcium normalization was defined as the achievement of albumin-corrected serum calcium ≤10.5 mg/dL (2.62 mmol/L) and maintenance at this level. Median time to calcium normalization was 2 months (IQR: 1.3-2.9 months). At one-month follow-up, seven of 22 patients (31.8%) achieved normalized calcium; at two-month follow-up, 14 of 22 patients (63.6%) achieved normalization; at three-month follow-up, 20 of 22 patients (90.9%) achieved normalization; and at six-month follow-up, all 22 patients (100%) achieved normalization with a mean calcium of 9.7±0.3 mg/dL. The temporal pattern of calcium normalization is detailed in Table 4.

**Table 4:** Temporal calcium normalization pattern during anti-tuberculosis therapy Calcium normalization is defined as albumin-corrected serum calcium ≤10.5 mg/dL (2.62 mmol/L) and maintenance at this level. Cumulative normalization represents the percentage of patients achieving and maintaining normalized calcium at each time point. All 22 patients with baseline hypercalcemia were assessed at each time point. Median time to complete normalization: 2 months (IQR: 1.3-2.9 months). Corrected serum calcium was calculated using the formula: corrected calcium=serum calcium+0.8×(4.0−albumin). "-" means not applicable; no patients in normalized state at baseline, no patients hypercalcemic at 6 months

Time point	Patients assessed, n	Cumulative patients normalized, n (%)	Corrected calcium (mg/dL) in normalized patients, mean±SD	Corrected calcium (mg/dL) in remaining hypercalcemic patients, mean±SD
Baseline	22	0 (0)	-	11.0±0.5
1 month	22	7 (31.8)	10.2±0.2	10.6±0.4 (n=15)
2 months	22	14 (63.6)	10.1±0.2	10.3±0.3 (n=8)
3 months	22	20 (90.9)	10.1±0.2	10.1±0.2 (n=2)
6 months	22	22 (100)	9.7±0.3	-

Clinical outcomes and complications

Treatment outcomes analysis revealed a high success rate, with 93 patients (93%) achieving treatment success according to standard criteria (bacteriological conversion at two months and clinical improvement with radiological resolution). Mortality occurred in two patients (2%) due to complications unrelated to hypercalcemia (one due to acute myocardial infarction, one due to severe nosocomial pneumonia), and treatment failure was documented in five patients (5%) due to non-adherence and loss to follow-up.

Among hypercalcemic patients specifically, interventions related to calcium management were required in two patients (9.1% of hypercalcemic patients) with symptomatic hypercalcemia, consisting exclusively of intravenous isotonic saline (0.9% sodium chloride) infusion (250 mL/hour for 4-6 hours). No patients required pharmacological intervention for hypercalcemia management (no glucocorticoids, no bisphosphonates, no calcitonin).

## Discussion

This study demonstrates that hypercalcemia occurs in 22% (95% CI: 14.6-31.6%) of patients with microbiologically confirmed pulmonary TB. Our findings align closely with reports from other international studies showing prevalences of 20% in Indian patients with TB [1], 27.5% in Nigerian patients [13], and 25% in Swedish cohorts [3]. The predominance of mild, asymptomatic hypercalcemia in our cohort (72.7% mild, 90.9% asymptomatic) mirrors findings from other contemporary series, emphasizing the importance of biochemical screening rather than symptom-based detection.

A critical finding of our study is the uniform suppression of PTH in all hypercalcemic patients (6.2±2.0 pg/mL, range 3.3-9.6 pg/mL), with 100% demonstrating values below the lower limit of the reference range (10 pg/mL). The combination of elevated calcium with suppressed PTH effectively excludes primary and secondary hyperparathyroidism, which together account for the majority of hypercalcemia cases in outpatient settings [5,6].

The finding of normal 25(OH)D levels (37.0±6.0 ng/mL) in our hypercalcemic cohort provides additional diagnostic clarity by excluding vitamin D intoxication and supporting the mechanism of extrarenal 1α-hydroxylase activity. This biochemical profile, elevated calcium, suppressed PTH, and normal 25(OH)D, is characteristic of granulomatous disorders, including TB and sarcoidosis, resulting from autonomous 1α-hydroxylase activity in activated macrophages within granulomas [8,9,14].

The finding of normal serum phosphate (3.1±0.5 mg/dL) in hypercalcemic patients provides additional diagnostic specificity. In primary hyperparathyroidism (a common alternative diagnosis), serum phosphate is typically significantly suppressed due to PTH-mediated urinary phosphate wasting. In contrast, granulomatous hypercalcemia with suppressed PTH demonstrates normal-to-mildly-elevated serum phosphate due to enhanced renal phosphate reabsorption in the absence of PTH-mediated urinary phosphate wasting.

Most importantly, this study directly measured serum calcitriol (1,25-dihydroxyvitamin D or 1,25(OH)₂D) levels, which were uniformly elevated (78.5±15.2 pg/mL; reference range 15-65 pg/mL), with all 22 hypercalcemic patients demonstrating values above the upper limit of normal. This finding directly confirms the hypothesized pathophysiological mechanism of extrarenal 1α-hydroxylase (CYP27B1 enzyme) activity in activated macrophages within granulomatous lesions. The degree of calcitriol elevation correlated with hypercalcemia severity (mild hypercalcemia: mean calcitriol 72.8±12.1 pg/mL; moderate hypercalcemia: mean calcitriol 91.3±14.7 pg/mL; p=0.008), supporting a dose-response relationship between calcitriol production and calcium elevation.

A key distinguishing feature of our study is the direct measurement of serum calcitriol by LC-MS/MS in all hypercalcemic patients. While previous TB hypercalcemia investigations have focused on PTH and 25(OH)D, we simultaneously measured all four parameters, including calcitriol. This comprehensive approach allowed us to directly confirm the granulomatous mechanism rather than infer it.

Our identification of disseminated TB (multivariate adjusted OR: 2.4, 95% CI: 1.1-5.3, p=0.028) and CKD (multivariate adjusted OR: 3.8, 95% CI: 1.3-11.1, p=0.015) as independent risk factors for hypercalcemia provides important insights for targeted surveillance strategies. Disseminated TB likely increases hypercalcemia risk through greater granulomatous burden across multiple organ systems, resulting in enhanced total 1α-hydroxylase activity and calcitriol production. Among our 17 patients with disseminated TB, seven (41.2%) developed hypercalcemia versus 10 of 83 patients (12.0%) without disseminated disease. In contrast to disseminated TB's mechanism involving increased granulomatous burden, the association between CKD and hypercalcemia likely reflects a distinct dual mechanism. Granulomatous macrophages produce calcitriol autonomously and independently of parathyroid hormone status, while simultaneously, reduced glomerular filtration impairs renal calcium excretion capacity. Consequently, TB patients with concomitant CKD experience compounded hypercalcemia risk through both autonomous calcitriol synthesis and impaired renal clearance. Among our nine patients with CKD, five (55.6%) developed hypercalcemia versus 17 of 91 patients without CKD (18.7%), supporting this mechanistic insight. 

The universal normalization of calcium levels with standard anti-TB therapy, achieved in all patients by six months with a median time of 2 months, confirms the self-limiting nature of TB-associated hypercalcemia and supports conventional treatment approaches. Prior studies have documented that anti-TB medications, particularly rifampicin and isoniazid, facilitate calcium normalization through restoration of normal vitamin D metabolism in granulomatous lesions and suppression of macrophage 1α-hydroxylase activity [15]. Our normalization timeline is consistent with reports from diverse geographic settings [3,13], suggesting that the natural history of treatment response is relatively uniform across populations.

The observation that only 9.1% of hypercalcemic patients required intervention (limited to intravenous fluids for two symptomatic patients) confirms that severe, symptomatic hypercalcemia remains uncommon in TB and is generally manageable with supportive care alongside anti-TB therapy. No patient required specific pharmacological intervention for hypercalcemia management (such as glucocorticoids or bisphosphonates).

Given that most hypercalcemic patients in our cohort were asymptomatic and required no pharmacologic intervention, these findings suggest that calcium screening with subsequent biochemical evaluation may be considered selectively in adults with pulmonary tuberculosis, particularly in those with disseminated disease or chronic kidney disease, while recognizing that this approach requires confirmation in larger, prospective studies.

This study has several limitations. The retrospective, single-center design may limit generalizability to other populations and healthcare settings. A retrospective approach was selected because comprehensive calcitriol testing is not routinely performed in all patients with tuberculosis in real-world clinical practice, making prospective systematic assessment logistically challenging. Information bias may arise from retrospective documentation, as measurements were recorded during routine clinical care rather than per a standardized study protocol, potentially introducing inconsistency in timing and completeness of biochemical evaluation.

The small number of hypercalcemic cases (n=22) limits statistical power for multivariable analysis and approaches the threshold for potential overfitting; risk factor estimates require validation in larger cohorts. Our exclusion criteria (MDR/XDR-TB patients and those receiving vitamin D or calcium supplementation) introduce selection bias and may limit applicability to these subgroups; conversely, exclusion of MDR/XDR-TB patients may have underestimated hypercalcemia prevalence if drug-resistant disease carries a higher granulomatous burden.

Potential confounding factors, including dietary calcium intake, sunlight exposure patterns, genetic polymorphisms affecting vitamin D metabolism, and seasonal variation, were not systematically assessed. Long-term follow-up beyond six months was not performed to evaluate potential late recurrence or long-term skeletal consequences of hypercalcemia exposure.

Despite these limitations, the study population reflects routine clinical practice in a tuberculosis-endemic region, and the observed biochemical patterns and treatment responses are likely applicable to similar high-burden settings where granulomatous tuberculosis remains common.

Future multicenter prospective studies incorporating comprehensive vitamin D metabolite profiling, including calcitriol, urinary calcium, and phosphate measurements, systematic assessment of clinical outcomes stratified by hypercalcemia severity, genetic analysis of vitamin D metabolism polymorphisms, and long-term follow-up extending beyond six months are warranted to further elucidate the pathophysiology, risk stratification, optimal management strategies, and long-term sequelae of TB-associated hypercalcemia.

## Conclusions

Hypercalcemia represents a common metabolic complication affecting approximately 22% of patients with pulmonary TB, predominantly manifesting as mild, asymptomatic elevations that resolve completely with standard anti-TB therapy. The characteristic biochemical profile of suppressed PTH with normal 25-hydroxyvitamin D, normal serum phosphate, and markedly elevated 1,25-dihydroxyvitamin D (calcitriol) confirms the extrarenal mechanism of calcitriol synthesis mediated by activated macrophages within granulomatous lesions and effectively distinguishes granulomatous from parathyroid-mediated hypercalcemia. 

Disseminated TB and CKD emerge as significant independent risk factors warranting enhanced calcium surveillance. These findings support routine calcium screening in patients with TB, particularly those with identified risk factors, with PTH and 25-hydroxyvitamin D measurement in hypercalcemic individuals to confirm the characteristic biochemical pattern and exclude alternative etiologies. Serum phosphate and calcitriol measurement should be incorporated into hypercalcemia evaluation protocols to strengthen differential diagnosis and confirm the granulomatous mechanism.
